# Detection of genomic regions associated with tiller number in Iranian bread wheat under different water regimes using genome-wide association study

**DOI:** 10.1038/s41598-020-69442-9

**Published:** 2020-08-20

**Authors:** Sayedeh Saba Bilgrami, Hadi Darzi Ramandi, Vahid Shariati, Khadijeh Razavi, Elahe Tavakol, Barat Ali Fakheri, Nafiseh Mahdi Nezhad, Mostafa Ghaderian

**Affiliations:** 1 Department of Plant Molecular Biotechnology, National Institute of Genetic Engineering and Biotechnology (NIGEB), Tehran, Iran; 2 College of Agronomy and Biotechnology, Southwest University, Beibei, 400715 Chongqing China; 3 Department of Molecular Physiology, Agricultural Biotechnology Research Institute of Iran, Research, Education and Extension Organization (AREEO), Karaj, Iran; 4 Department of Plant Production and Genetics, Shiraz University, Shiraz, Iran; 5 Department of Plant Breeding and Biotechnology, Faculty of Agriculture, University of Zabol, Zabol, Iran; 6 Department of Plant Breeding and Biotechnology, Faculty of Agriculture, Isfahan University of Technology, Isfahan, Iran

**Keywords:** Plant sciences, Plant breeding, Plant genetics, Plant stress responses, Genetics, Agricultural genetics, Genetic association study, Genotype, Plant breeding, Plant genetics

## Abstract

Two of the important traits for wheat yield are tiller and fertile tiller number, both of which have been thought to increase cereal yield in favorable and unfavorable environments. A total of 6,349 single nucleotide polymorphism (SNP) markers from the 15 K wheat Infinium array were employed for genome-wide association study (GWAS) of tillering number traits, generating a physical distance of 14,041.6 Mb based on the IWGSC wheat genome sequence. GWAS analysis using Fixed and random model Circulating Probability Unification (FarmCPU) identified a total of 47 significant marker-trait associations (MTAs) for total tiller number (TTN) and fertile tiller number (FTN) in Iranian bread wheat under different water regimes. After applying a 5% false discovery rate (FDR) threshold, a total of 13 and 11 MTAs distributed on 10 chromosomes were found to be significantly associated with TTN and FTN, respectively. Linked single nucleotide polymorphisms for IWB39005 (2A) and IWB44377 (7A) were highly significantly associated (FDR < 0.01) with TTN and FTN traits. Moreover, to validate GWAS results, meta-analysis was performed and 30 meta-QTL regions were identified on 11 chromosomes. The integration of GWAS and meta-QTLs revealed that tillering trait in wheat is a complex trait which is conditioned by the combined effects of minor changes in multiple genes. The information provided by this study can enrich the currently available candidate genes and genetic resources pools, offering evidence for subsequent analysis of genetic adaptation of wheat to different climatic conditions of Iran and other countries.

## Introduction

Bread wheat (*Triticum aestivum* L., genomes AABBDD, 2n = 6x = 42), is a major cereal crop, supplying 20% of the total energy and protein of the world’s diet^1^. Its production and productivity, especially in arid and semiarid regions such as Iran, are considerably constrained by extreme drought and heat stresses. Breeding for grain yield is the final step to produce stress-tolerant crop plants, since grain yield is a complex trait with low heritability, which is controlled by multiple genes and is affected by a lot of environmental factors, other traits such as yield components can be employed to overcome the limitations. Tillering is a crucial factor for wheat yield because of its involvement in grain weight and grain number determination. Moreover, it is a determinant of grain yield, since the tiller number is key in regulating competition between the tillers and the main shoot for assimilating supply^2,3^. The plant architecture and its tillering system impact many factors, including the light-harvesting and yield potential of the plant, the flowering and grain set, and last but not the least, the plant’s reproductive success. Therefore, genetic elucidation of tiller numbers at various stages of plant growth has become a major focus of wheat breeding research programs^4^.

Tiller number is inherited quantitatively in most cases and is affected by soil fertility and environmental factors, especially temperature and day length. Although four single genes (tin1, tin2, tin3, ftin) responsible for tiller inhibition are mapped on the wheat chromosomes 1A, 2A and 3A^5,6^, since this fundamentally principal trait is polygenic, most of the underlying variation for tillering was found to be controlled by quantitative trait loci (QTL)^7,8^. Recently, QTL mapping and genome-wide association studies (GWAS) have become two key approaches to understanding the genetic bases and dissection of complex genes and controlling important features such as tillering traits. In spite of the success of QTL mapping in detecting QTL, the genetic variation of the population has been so far limited only to the genomes of the parents. Additionally, the genetic markers for the identified QTL can not necessarily be transferred to other populations. Such transferability would be desirable, given that genome-wide association study is a high-resolution and cost-effective method that depends on high-density marker and trait associations, employing genetically varied populations like landraces, elite breeding lines, and cultivars to elucidate the genetic architecture of agronomic traits^9^.

In addition to the benefits of GWAS to QTL mapping, the meta-analysis as a statistical technique was developed to combine consensus loci from many individual QTL studies for any number of traits into a single dataset to identify most likely position and confidence interval (CI) of QTL regions^10^. This method has been used to determine consensus regions of the genome across multiple QTL studies for their effects and consistency across different genetic backgrounds and environments, also to refine and confirm QTL positions on a consensus map via mathematical models. Reducing the CI of the MQTLs in comparison with QTLs is another indispensable perks of MQTL analysis^11–15^. More recently, a number of studies have applied the QTL meta-analysis method to different traits in wheat, including root morphological traits^13,16^, grain traits^11^, fusarium head blight resistance^14,17^, adaptation to drought and heat stress^18^ and leaf rust resistance^19^.

In the past, wheat improvement has been based on selections from landraces, followed by crossing between landraces, introducing varieties, and finally crossing between elite varieties^20^. Iranian bread wheat landraces possess rich genetic diversity, with a large number of rare alleles or potentially new alleles so that they can display high levels of resistance to many different abiotic stresses such as drought. However, most Iranian germplasm genotypes have not yet been characterized or utilized in modern plant breeding^21^. Hence, the examination of genetic variations and the distinction among Iranian wheat landraces and cultivars will be of great value. Not only may it identify new sources of resistance to drought and other stressors, but it may increase the biodiversity of the materials available for wheat breeding. Moreover, novel alleles will be identified that may be of value for Iranian wheat geneticists and breeders.

While some GWAS studies have used SNPs to examine the association of some traits in wheat under water-limited conditions, to the best of our knowledge, no GWAS study identifying the association of different agronomic traits in Iranian wheat under drought stress has been reported. Therefore, the objective of the present study, using 15 K SNP array markers, is to identify the structure of population and genome-wide marker-trait associations of Iranian wheat total tiller number (TTN) and fertile tiller number (FTN) under different water regimes, and environmets with breeding system design such as drought-tolerance improvement. In this regard, we used a multi locus GWAS model named fixed and random model circulating probability unification (FarmCPU) method as a new and more efficient recently developed method^22,23^. Furthermore, to confirm and strengthen the results of MTAs, QTL meta-analysis was conducted to identify wheat genome regions that are consistently associated with tiller traits.

## Results

### Statistical description of phenotypic data

A combined analysis of variance (ANOVA) was performed and descriptive statistics including the min, max and mean of the tiller number traits were estimated (Supplementary Tables S1 and S2). ANOVA revealed significant differences for both genotype (G) and location (L). A significant difference was observed between the two irrigation regimes (S) in terms of tiller traits in three locations or in fact environments (Shahed university field for two years and NIGEB field for one year) and in their interaction. The effects of G × S, G × L, and G × S × L were not significant for total tiller number and fertile tiller number (Supplementary Table S2). Also, the data for the two irrigation regimes in all three locations, (summarized in Supplementary Fig. S1 and Supplementary Table S1) were separately analyzed to generate BLUEs of genotype performance within each environment, for use in the subsequent analysis. Heritability of 48 and 43% was estimated for TTN and FTN, respectively, under all conditions (Supplementary Table S2). These traits were estimated to have moderate heritability (20% < *h*^2^ < 50%).

The means of TTN across 24 wheat landraces plus 70 wheat cultivars in the six environments ranged from 6.11 in NIGEB drought condition in 2015 to 15.77 in the normal environment in research field at the Shahed university in 2014 (Supplementary Table S1). BLUEs across all normal irrigation conditions ranged from 8.45 for Hirmand cultivar to 15.49 for Ebrahim Abad Arak landrace with a mean value of 11.16. BLUEs across all drought conditions ranged from 7.67 for the Mihan cultivar to 14.08 for the Ebrahim Abad Arak landrace, with a mean value of 10.54. The BLUEs across all the six environments ranged from 8.26 for the Mihan cultivar to 14.85 for the Ebrahim Abad Arak landrace, with a mean value of 10.86.

For FTN, means in the six field environments ranged from 4.22 in the NIGEB drought condition to 12.83 in the normal condition at the Shahed farm during 2013 to 2014 (Supplementary Table S1). The mean value of the best linear unbiased estimations was 8.89 and ranged across all normal irrigation conditions from 6.66 for the Hirmand variety to 12.99 for the Khoram Abad landrace. Moreover, the mean values for the all drought conditions ranged from 6.15 for the Mihan variety to 11.24 for the Ebrahim Abad Arak landrace, with a mean value of 8.16. BLUEs across all six environments ranged from 6.74 for the Pishgam cultivar to 11.96 for the Ebrahim Abad Arak genotype, with a mean value of 8.53. The correlations between total tiller number and fertile tiller number, and between grain yield and phenological charecters (BLUEs values) are shown in Supplementary Fig. S2. TTN and FTN had significant positive correlations ranging more than 0.8 in the normal, drought and all environments. There were significant and positive correlations between total tiller number and phenological traits, including days to heading (0.22^*^), days to flowering (0.23^*^), days to maturity (0.26^*^) in normal and all environments and also between TTN and days to maturity (0.21^*^) in drought condition (Supplementary Fig. S2). The FTN character showed insignificant correlations with the phenological characters. Furthermore, between FTN and grain yield (0.21^*^) there was significant correlation in normal condition.

### Analysis of SNP markers

Out of the 13,006 SNPs in the Illumina iSelect 15 K SNP assay, a total of 10,054 SNPs were polymorphic among the 92 wheat genotypes. Eliminating the markers with minor allele frequencies (MAF < 0.1), the dataset was narrowed to 6,349 SNPs mapped to 21 wheat chromosomes; this dataset was later used for GWAS analysis. The analysis detected 12,698 alleles (two alleles per SNP locus). About 60% of the SNPs (those with MAFs > 0.20), were then chosen as normal allele frequency markers, while 8% of SNPs displayed approximately equal allele frequencies (MAFs ~ 0.1). The physical positions of 6,349 SNP were determined based on the Chinese Spring reference assembly IWGSC RefSeqv1.0 (https://urgi.versailles.inra.fr/). Estimated PIC values and overall SNP diversities, for these 6,349 SNP markers ranged from 0.263 to 0.321 with an average of 0.306, where; the PIC values for about 70% exceeded 0.20. Among the 21 chromosomes, chromosome 2B had the maximum number of markers (*n* = 599), followed by chromosome 5B (*n* = 491), while chromosome 4D had the fewest loci (*n* = 23), among sub-genomes of A, B, and D. The B sub-genome carried the most loci (*n* = 2,944), followed by A sub-genome (*n* = 2,351), and the D sub-genome (*n* = 1,054).

### Population structure

**Figure 1:**
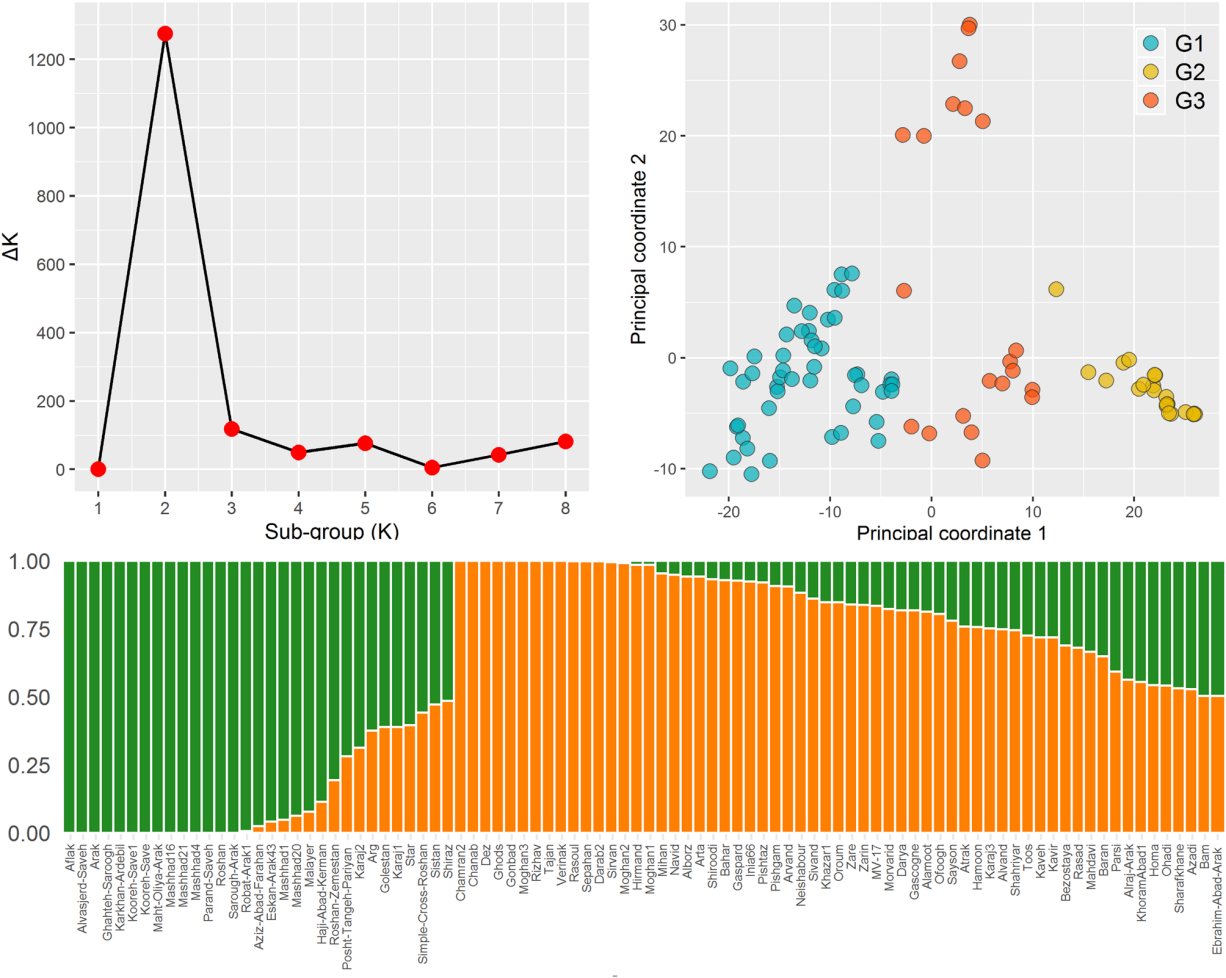
The structure plot of the 92 Iranian wheat cultivars and landraces identified by K = 2 using 6,349 SNPs. (**a**) Plot of the ad hoc statistic ΔK, which is ΔK tends to peak at the K value that corresponds to the highest hierarchical level of substructure. The modal value of this distribution is the true K, here two clusters. (**b**) Principal coordinate analysis (PCoA) plot of the first two PCo in 92 wheat genotypes including landrace (yellow), cultivar genotype (blue) and intermediate cultivar genotype (red). The first and second PCo respectively make up 19 and 6% of the total variation. (**c**) Population structure inference of the 92 Iranian wheat genotypes based on SNP marker, using STRUCTURE. Vertical bars display individual genotypes. The segment color length in each vertical bar depicts the proportion contributed by each of the two populations in the model to that individual.

Delta K (ΔK) values were determined using STRUCTURE to investigate the genetic diversity and population structure of the Iranian wheat panel and to classify the subgroups (K). The value of ΔK was plotted against the number of presumptive subgroups *K,* with the highest ΔK observed when *K* = 2 (Fig. 1a), indicating the existence of two subgroups in the genotype panel used in this study. Group I (designated as ‘landrace group’) contains 23 genotypes with 18 landraces and 5 cultivars; Group II (designated as ‘cultivar group’) consists of 48 cultivars, (Fig. 1c). Most cultivars that mixed with the landrace group—such as Bam, Azadi, Sistan, Shiraz, Homa, Ohadi, Karaj, and Golestan- had been originally chosen from Iranian landraces through permanent selection and purification during the breeding process (Supplementary Table S3). To examine the population structure of the wheat genotypes, a PCoA was carried out, with the first two principal coordinates explaining 19 and 6% of the total variability, respectively (Fig. 1b). The results of the analysis depicted two main sub-populations; the landraces genotypes (G2) were clearly separated from wheat cultivars (G1), while some cultivars were located in an intermediate group (G3).

### Marker-trait association analysis

In line with Kaler et al.^23^, The fixed and random model circulating probability unification (FarmCPU) provides a robust model for association mapping of complex traits in plants which can controls both false positives and false negatives and consistently identify a single significant SNP closest to these known published genes. Both computer simulation and real data analyses demonstrated that FarmCPU is the highly efficient method to reduce confounding issues arising due to kinship, population structure, multiple testing correction than MLM, stepwise regression, etc. Therefore, FarmCPU approch were used in this study to determine the marker-trait associations controlling TTN and FTN under both normal and stress conditions along with all environments using the best linear unbiased estimator (BLUEs) values calculated across the three locations of the experiment. Piepho et al.^24^ previously discovered that MTAs are more stable if BLUEs data are used in the absence of environmental effects.

**Figure 2:**
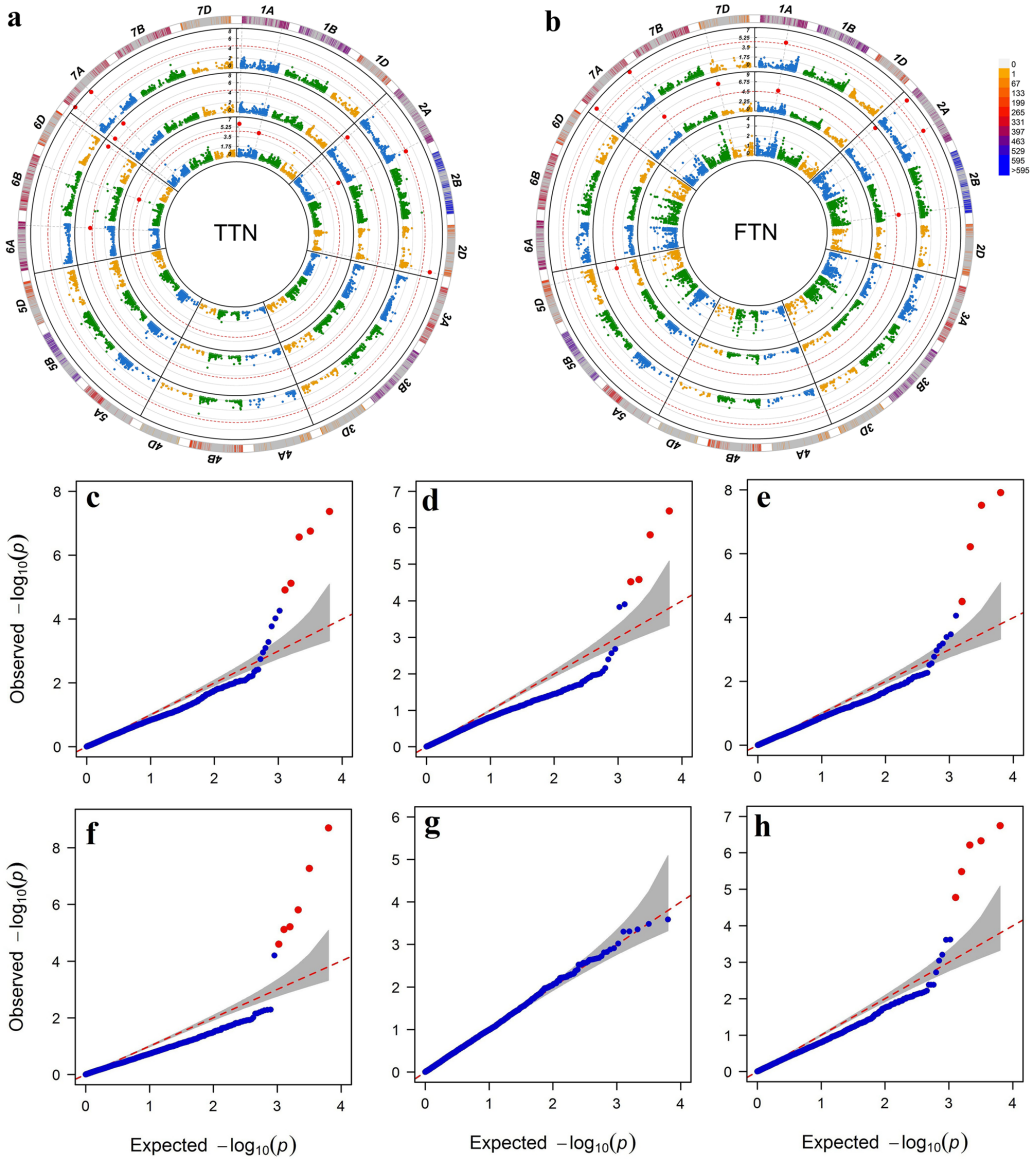
Circular-Manhattan plots and quantile–quantile plots for SNP significantly associated with tillering numbr under normal and drought stress conditions identified by genome-wide association study based on the fixed and radom model Circulating Probability Unification (FarmCPU). (**a**) SNP-GWAS associated with total tiller number (TTN) in BLUEs data (outer-most), normal (middle) and drought (inner-most) conditions. (**b**) SNP-GWAS associated with fertile tiller number (FTN) in BLUEs data (outer-most), normal (middle) and drought (inner-most) conditions. The dashed red line represents the false-discovery rate (FDR) threshold (FDR ≤ 0.05). SNPs marker that met this significant level are highlighted with red dots. QQ plot of genome-wide associations for (**c**) total tiller number under normal, (**d**) total tiller number under drought stress, (**e**) total tiller number in BLUEs data, (**f**) fertile tiller number under normal, (**g**) fertile tiller number under drought stress, (**h**) fertile tiller number in BLUEs data. For QQ plots, X-axis represents expected − log_10_ (*p-value*) and Y-axis is observed − log_10_ (*p-value*) of each SNPs. The physical positions of the SNP markers were determined based on the Chinese Spring reference assembly IWGSC RefSeqv1.0 (https://urgi.versailles.inra.fr/).

Significant MTAs (− log_10_ (*p*-value) ≥ 3.0) were identified for the traits in the three environments examined. All chromosomes carried the MTAs, except for chromosomes 3A, 3D, 4A, 4D and 5B. Of all significant SNPs, in the three environments examined, 25 and 22 were significantly associated with total tiller number and fertile tiller number, respectively, and among these, two SNPs were significantly associated with both traits (Supplementary Table S4). When FDR-correction was applied for multiple testing (*p-value* = 0.05), 24 MTAs remained significant for SNP markers (FDR ≤ 0.05) for both traits in the three environments (Table 1). The Manhattan and QQ plots are also, shown in Fig. 2. The efect of SNPs involved in individual MTAs ranged from − 0.36 to 0.85 for TTN-ALL, − 0.17 to 0.16 for TTN-N, − 0.29 to 0.34 for TTN-S, − 0.69 to 0.67 for FTN-ALL, − 1.22 to 0.76 for FTN-N (Table1). For TTN and FTN, more than one SNPs were found to be associated under both normal and stress conditions as well as all environments, i.e., IWA5084, IWA4483 in FTN-ALL and FTN-N; IWB44155 in TTN-ALL, TTN-N and TTN-S; IWB44377, in TTN-ALL, TTN-N and FTN-ALL; IWB39005 in FTN-ALL, FTN-N and TTN-N. No significant MTAs for FTN in the drought-stress condition were detected at FDR threshold (Supplementary Table S4).

**Table 1:** Summary of significant SNP markers identified by GWAS mapping associated with tillering number traits.

Trait	Env	SNP	Chromosome	Position (bp)	− log_10_(*p-value*)	Allele	MAF^a^	FDR	Effect
FTN	Normal	IWA5084	1A	369,697,871	5.12	A/G	0.196	0.00961	− 0.565
FTN	Normal	IWB39005	2A	36,632,023	8.69	A/G	0.136	0.00001	− 1.224
FTN	Normal	IWB28961	2B	713,676,010	5.21	A/G	0.147	0.00961	0.760
FTN	Normal	IWB11256	5D	489,775,906	4.60	C/T	0.136	0.02648	− 0.704
FTN	Normal	IWA4483	7A	692,340,211	5.81	C/T	0.272	0.00328	0.749
FTN	Normal	IWB19377	7D	58,491,640	7.28	A/G	0.120	0.00017	− 1.041
FTN	All	IWA5084	1A	369,697,871	4.77	A/G	0.196	0.02129	− 0.388
FTN	All	IWB36728	2A	500,529,738	5.49	C/T	0.179	0.00515	− 0.618
FTN	All	IWB39005	2A	36,632,023	6.33	A/G	0.136	0.00130	− 0.693
FTN	All	IWB44377	7A	33,364,868	6.21	C/T	0.473	0.00130	0.492
FTN	All	IWA4483	7A	692,340,211	6.74	C/T	0.272	0.00115	0.677
TTN	Normal	IWB39005	2A	36,632,023	5.11	A/G	0.136	0.01213	− 0.133
TTN	Normal	IWB25250	6A	574,486,383	4.90	C/T	0.250	0.01574	− 0.115
TTN	Normal	IWA1406	6D	463,447,036	6.75	A/G	0.190	0.00056	− 0.174
TTN	Normal	IWB44155	7A	323,740,318	7.36	C/T	0.158	0.00027	0.161
TTN	Normal	IWB44377	7A	33,364,868	6.57	C/T	0.473	0.00057	0.107
TTN	Stress	IWB53633	1A	517,488,008	4.58	A/G	0.361	0.05061	0.271
TTN	Stress	IWB74344	1A	1,208,254	5.81	C/T	0.117	0.00492	0.346
TTN	Stress	IWA6592	2A	715,301,715	6.46	C/T	0.239	0.00221	0.287
TTN	Stress	IWB25244	6B	626,453,853	4.50	G/T	0.106	0.05061	− 0.295
TTN	All	IWB9024	2A	747,144,155	4.51	C/T	0.413	0.05001	− 0.369
TTN	All	IWB55568	2D	641,963,416	6.22	A/G	0.473	0.00128	0.558
TTN	All	IWB44155	7A	323,740,318	7.51	C/T	0.158	0.00010	0.857
TTN	All	IWB44377	7A	33,364,868	7.91	C/T	0.473	0.00008	0.607

The FDR corrected threshold (− log_10_ (*p-value*) ≥ 4.5) was used to identify significant SNP–trait associations. a: Minor allele frequency. TTN: Total tiller number; FTN: Fertile tiller number; Normal: BLUEs value across all normal irrigation conditions; Stress: BLUEs value across all drought stress conditions; All: BLUEs value based on 6 environmets under normal and drought stress conditions.

**Figure 3:**
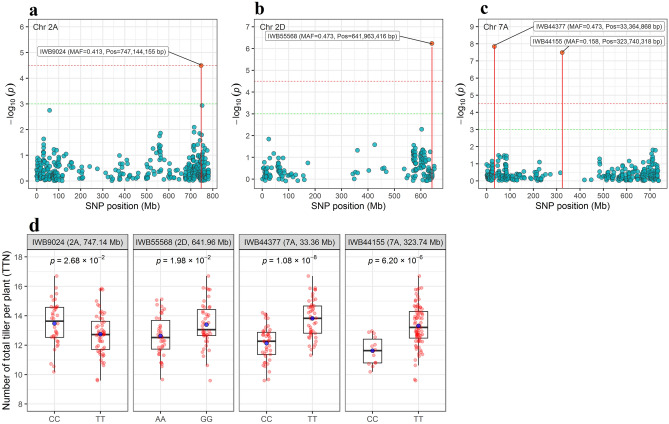
GWAS-derived Manhattan plots showing significant SNPs associated with the total tiller number (BLUEs values) on chromosome 2A (**a**), 2D (**b**) and 7A (**c**) using FarmCPU method. The horizontal dashed red line indicates FDR significance threshold of *P* = 0.05. (**d**) The box plots of the allele effects for the four SNPs associated with total tiller number.

**Figure 4:**
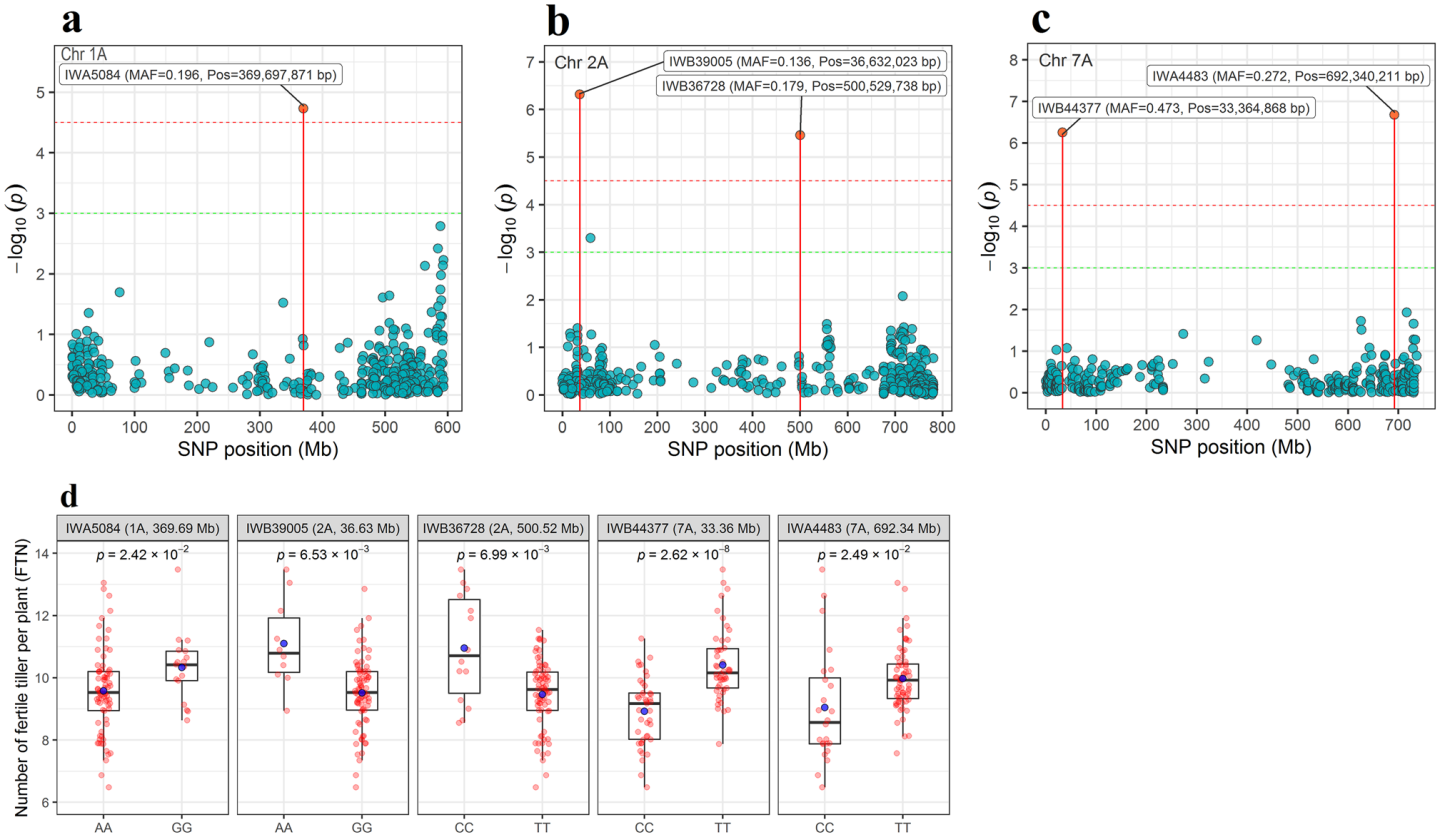
GWAS-derived Manhattan plots showing significant SNPs associated with the fertile tiller number (BLUEs values) on chromosome 2A (**a**), 2D (**b**) and 7A (**c**) using FarmCPU method. The horizontal dashed red line indicates FDR significance threshold of *P* = 0.05. (**d**) The box plots of the allele effects for the five SNPs associated with fertile tiller number.

SNPs associated with two phenotypic traits; TTN (Fig. 3) and FTN (Fig. 4) were used to show the allelic effects on the BLUEs values. Eight high significant SNPs on chromosome 1A, 2A, and 7A associated with TTN and FTN were assessed for their allelic effects on the BLUEs values. Among these SNPs, only IWB44377 on chromosome 7A at position 33,364,868 bp was associated with TTN and FTN. Three significant SNPs; IWB9024 (2A: 747,144,155 bp), IWB55568 (2D: 641,963,416 bp) and IWB44155 (7A: 323,740,318 bp) were only associated with TTN, while for FTN four significant SNPs, IWB5084 (1A: 369,697,871 bp), IWB39005 (2A: 36,632,023 bp), IWB36728 (2A: 500,529,738 bp) and IWA4483 (7A: 692,340,211 bp).

### QTL distribution and over-view index

**Figure 5:**
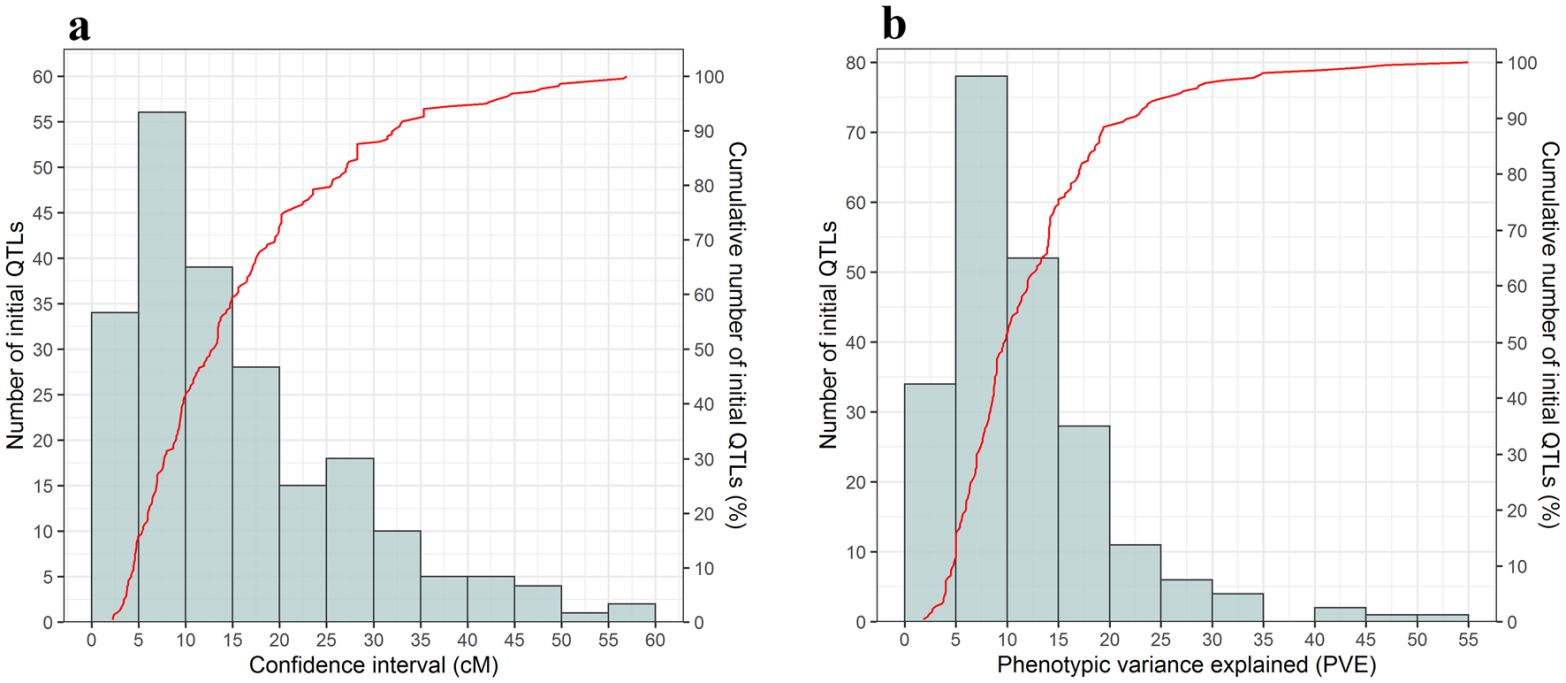
Distribution of initial QTLs for tillering number. (**a**) Frequency distribution of initial QTLs density based on different levels of 95% confidence interval, (**b**) Distribution of phenotypic variance explained (PVE) for each initial QTLs.

**Figure 6:**
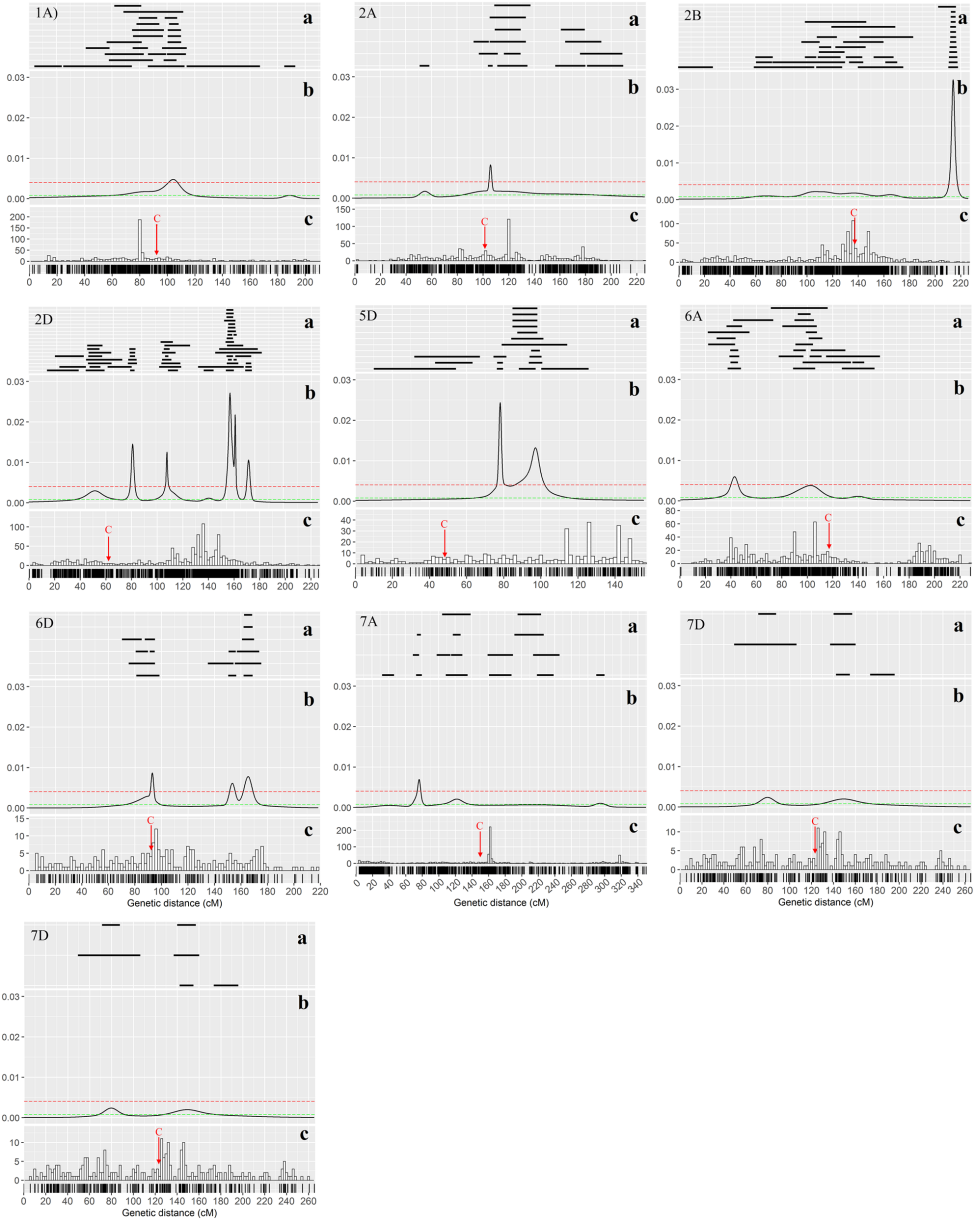
Diagrams show the different features that were drawn using the ggplot2 package in R environment. (**a**) Distribution of initial QTLs on each chromosome of wheat (black lines). (**b**). Probability density computed as ‘QTL-overview index’^25^. (**c**) Distribution and density of marker on the consensus genetic map. The red dashed line with the value of 0.00106 indicates the high-value threshold. The red color arrow indicates the centromeric region of the chromosome.

The reported QTLs on ten chromosomes (1A, 2A, 2B, 2D, 5D, 6A, 6B, 6D, 7A, and 7D) for total tiller number and fertile tiller number in wheat were collected from studies published since 2002 (Table 2). Information concerning QTLs including population types, flacking markers, the log of odds ratio (LOD score) and proportion of phenotypic variance explained by the QTL (*R*^*2*^) was extracted from 217 QTLs from the literatures. Among the 217 initial QTLs, 140 (65%) and 77 (35%) QTLs were found for TTN and FTN, respectively. The 95% confidence interval (CI) ranged from 2.26 to 56.94 cM, with an average of 15.90 cM. About 91 (42%) of the collected QTLs had a CI lower than 10 cM, and 158 QTL (73%) had a CI lower than 20 cM (Fig. 5a). The proportion of phenotypic variance explained (PVE) by the initial QTLs varied from 2.2 to 55.4% with an average of 12.15% (Fig. 5b). To identify the genomic regions most commonly associated with the number of total tillers and fertile tiller, the probability density computed as a QTL-overview index^25^ for each 0.5 cM-long segment on the consensus map (Fig. 6, Supplementary Table S5). A total of 34 peaks were obtained (Fig. 6a), of which the density curve for 27 peaks was much higher than the average value, indicating the presence of “real QTLs” (Fig. 6b, Fig. 6c). Additionally, 15 peaks exceeded a high-value threshold estimated as five times the mean value of the overview index. The number of overview peaks exceeding the average threshold ranged from two peak in chromosome 1A and 7D to seven peaks in chromosome 2D (Fig. 6).

**Table 2:** Bibliography of QTL studies involving total tiller and fertile tiller number in wheat used for meta-analysis.

Ref. No	Population			QTL		References
	Parents	Size	Type	Marker type	Projected number	
1	RAC875 × Kukri	368	DH	DArT, SSR	7	^63^
2	Kharchia65 × HD2009	114	RIL	SSR	1	^64^
3	Opata85 × W7984	114	RIL	SSR	7	^65^
4	Iran#49 × Yecora Rojo	168	RIL	SSR	3	^66^
5	Excalibur × Kukri	192	DH	DArT, SSR	2	^67^
6	Berkut × Krichauff	152	DH	DArT, SSR	8	^68^
7	Weebill × Bacanora	105	RIL	DArT, SSR, KASP	7	^69^
8	ND3338 × JD6	203	DH	SNP	19	^70^
9	Fukuho-kumogi × Oligoculm	107	DH	AFLP, SSR	4	^71^
10	CN18 × T1208	371	RIL	SSR	3	^72^
11	Flair × XX86	111	BC	SSR	1	^73^
12	Nanda2419 × Wangshuibai	230	RIL	SSR	2	^74^
13	WL711 × PH132, Opata85 × W7984	110	RIL	SSR	12	^75^
14	Huapei3 × Yumai57	168	DH, IF_2_	SSR, EST-SSR	17	^76^
15	Opata85 × W-7984	111	RIL	SSR	7	^77^
16	20,828 × Chuannong16	199	RIL	SNP	3	^78^
17	Q1028 × Zhengmai9023	186	RIL	DArT, SSR	2	^79^
18	Reeder × Conan	91	RIL	DArT, SSR	8	^80^
19	Xiaoyan54 × Jing411	142	RIL	SSR	4	^81^
20	CN18 × T1208	371	RIL	SNP	13	^7^
21	McNeal × Thatcher	160	RIL	DArT, SSR	3	^82^
22	Hanxuan10 × Lumai14	120	DH	AFLP, SSR, EST-SSR	16	^83^
23	Lovrin10 × Chinese Spring	92	DH	SSR	8	^84^
24	Chuanmai42 × Chuannong16	127	RIL	SSR, SRAP	6	^85^
25	Line3228 × Jing 4,839	237	F_2:3_	SSR	3	^86^
26	H461 × CN16	249	RIL	SNP	30	^42^
27	UIP × SYC	110	DH	SNP	4	^8^
28	NAUH167 × Wangshuibai	93	RIL	SSR	9	^87^
29	Chuan35050 × Shannong483	131	RIL	DArT, SSR	3	^88^
30	Attila × CDC Go	167	RIL	SNP	5	^89^

### QTL meta-analysis

To verify the significant SNP markers identified by GWAS on chromosomes 1A, 2A, 2B, 2D, 5D, 6A, 6B, 6D, 7A and 7D, reported QTLs associated with the total tiller and fertile tiller number (Table 2) were collected and projected into a consensus genetic map to integrate overlapping loci as consensus QTLs. Using BioMercator 4.2 software, meta-analysis was conducted based on the 217 initial QTLs and a total of 30 meta-QTLs were identified (Table 3). Only meta-QTLs comprising more than two QTLs from different studies were used for further analysis. Table 3 presents the information about each meta-QTL, including position (cM), 95% CI (cM), mean phenotypic variance explained (PVE), flanking markers, number of initial QTLs and number of studies reporting on that QTLs. The meta-QTLs identified on each chromosome varied from one for chromosome 6B to five on chromosome 2D. The mean of phenotypic variance explained by each meta-QTL ranged from 3.84% (MQTL7D-1) to 36.0% (MQTL2D-2), and the overall average was 11.20%. The 95% genetic CI for the meta-QTLs ranged between 0.12 (MQTL2B-5) and 16.33 (MQTL5D-1) cM, with an average of 5.04 cM, representing a reduction of more than 68.3% from those observed in the original QTLs (range = 2.3–56.9 cM; average = 15.9 cM). The physical intervals of the meta-QTLs varied from 1.47 (MQTL2D-5) to 94.0 (MQTL5D-1) Mb, and the interval of 11 meta-QTLs were less than 10 Mb (Table 3).

**Table 3:** Results of meta-analysis of QTLs controlling total tiller and fertile tiller number in wheat.

Meta QTL	Chr	MQTL position (cM)	95% confidence interval (cM)	Genomic position (Mb)	Mean initial QTL confidence interval (cM))	Coefficient of reduction in CI from mean original QTLs to MQTL	Mean R^2^ for the original QTL	Flanking markers	Number of initial QTLs	Number of experiments	Number of genes laying at the MQTL interval
MQTL1A-1	1A	72.43	5.94	16.44–19.81	21.98	3.7	9.19	Xgpw2005-Xwmc95	12	8	18
MQTL1A-2	1A	103.8	0.93	232.73–236.4	18.5	19.89	11.6	Xwmc183-Xgpw2045	12	6	7
MQTL2A-1	2A	105.4	3.7	310.83–320.05	10.12	2.73	13.67	Xgwm473-Xwmc455	3	2	17
MQTL2A-2	2A	121.9	8.66	668.68–682.62	23.6	2.72	3.06	Xwmc261-Xgwm445	6	2	181
MQTL2A-3	2A	177.4	8.03	709.62–758.39	26.36	3.28	6.63	IWB43724.1-IWB39958	5	3	874
MQTL2B-1	2B	63.91	8.82	31.72–41.46	25.06	2.84	5.91	Xwmc25-wPt-5374	4	4	112
MQTL2B-2	2B	110.4	4.9	117.99–133.02	23.4	4.78	6.74	IWB58039- IWB73449	9	6	81
MQTL2B-3	2B	138.1	7.54	523.78–545.75	25.75	3.41	5.27	Xbarc128-IWB60084	5	4	138
MQTL2B-4	2B	163.8	7.67	686.04–708.2	25.55	3.33	4.97	wPt-8340-wPt-1646	4	4	234
MQTL2B-5	2B	214.1	0.12	793.02–796.68	5.86	48.87	12.8	Xbarc159-Xwmc356	13	2	83
MQTL2D-1	2D	45.52	3.75	37.53–48.17	15.48	4.13	11.12	Xwmc470-Xgwm484	11	7	86
MQTL2D-2	2D	73.46	1.7	74.94–87.76	6.39	3.76	36	IWB25847-P35/M48-4	7	2	175
MQTL2D-3	2D	98.31	1.78	553.72–570.41	9.52	5.35	13.88	Xcfd73-Xcfd62	9	5	212
MQTL2D-4	2D	142.6	1.22	632.08–647.5	9.67	7.93	12.62	Xwmc167-Xgwm382	19	4	325
MQTL2D-5	2D	155.5	0.26	648.12–649.59	5.6	21.53	14.84	XksuD23-XksuH16	3	2	29
MQTL5D-1	5D	49.6	16.33	302.38–396.42	33.28	2.04	18.4	XksuD30-Xcfd8	3	2	960
MQTL5D-2	5D	95.69	0.5	472.63–481.56	14.21	28.42	18.07	Xgwm212-Xcfd29	12	5	134
MQTL6A-1	6A	42.27	3.25	16.57–18.71	15.9	4.89	14.22	IWA6390- IWB6327	9	4	37
MQTL6A-2	6A	95.81	5.24	93.77–108.94	19.69	3.76	12.29	IWB12213-Xcfd190	8	4	161
MQTL6A-3	6A	105.4	5.24	467.05–500.51	18.82	3.59	9.12	Xgwm356-IACX5753	5	3	206
MQTL6A-4	6A	140	4.8	520.76–581.75	25.8	5.37	5.24	Xabc175-IWA4949	3	3	590
MQTL6B-1	6B	129.5	6.28	47.77–115.95	12.99	2.07	6.93	Xgwm508-IWB11358	4	3	489
MQTL6D-1	6D	83.82	7.21	62.08–86.72	14.63	2.03	6.34	Xcfd19-Xbarc202	3	2	231
MQTL6D-2	6D	92.64	3.33	115.02–117.36	9.68	2.91	14.1	Xbarc123-Xgpw304	3	2	21
MQTL6D-3	6D	153.2	3.97	419.65–434.81	10.32	2.6	11.13	Xgpw312-Xcsb112(Dhn5)	3	2	227
MQTL6D-4	6D	165.5	0.75	456.46–469.25	10.99	14.66	14.08	Xcfd5-Xcmwg684a	6	2	261
MQTL7A-1	7A	73.8	3.39	54.96–57.87	8.41	2.48	18.91	Xgwm60-IWB50066	4	3	47
MQTL7A-2	7A	119.5	6.68	83.94–117.08	20.07	3	11.03	IWB8251-Xcfa2174	5	3	291
MQTL7A-3	7A	207.3	11.25	518.18–576.77	28.93	2.57	4.19	Xwmc286-Xcfd20	6	3	430
MQTL7D-1	7D	79.56	8.11	38.72–40.56	27.6	3.4	3.84	Xwmc463-Xbarc352	2	2	14

**Figure 7:**
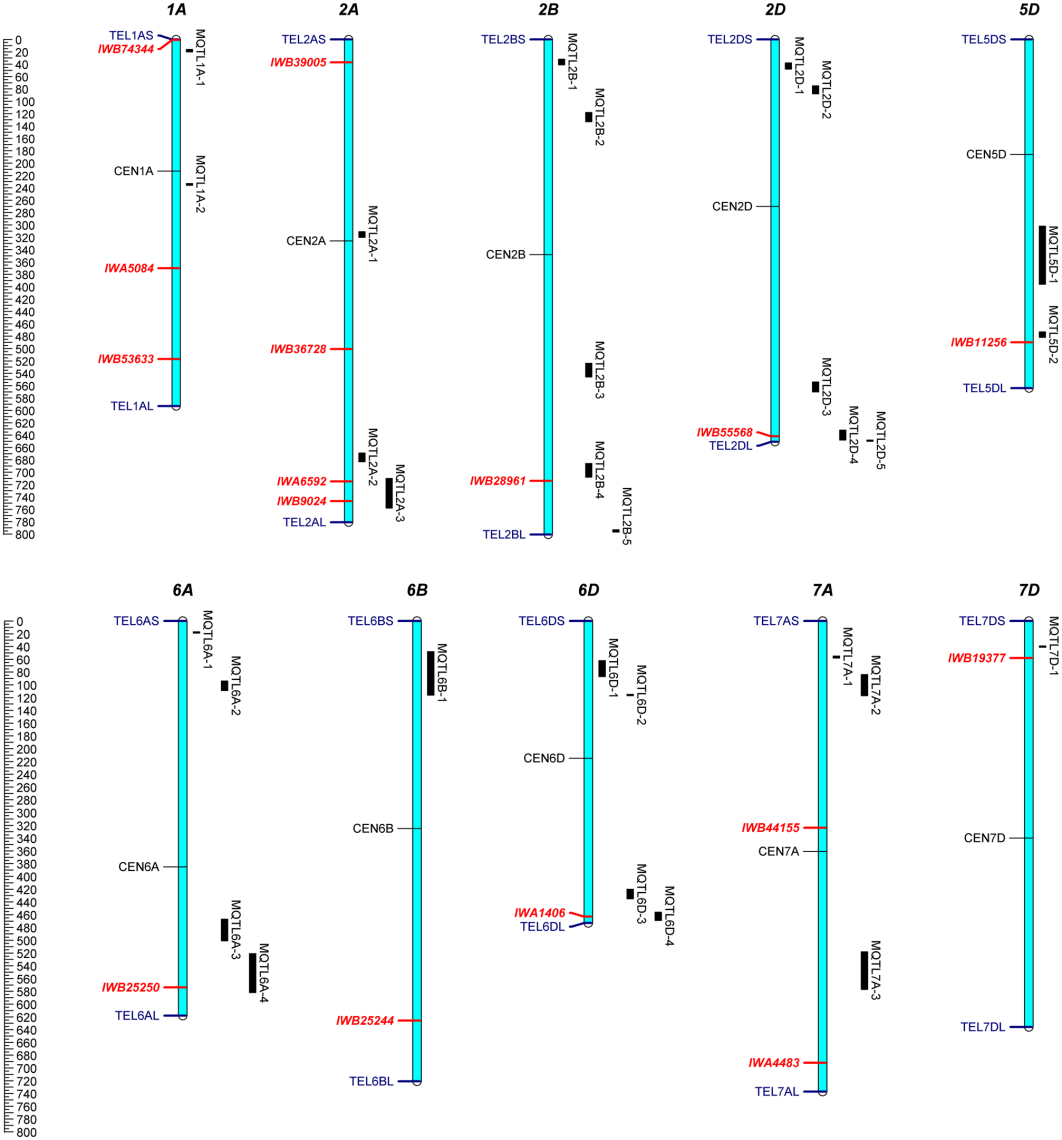
Genomic locations of detected MQTLs for tillering number that overlap with significant SNP markers from the GWAS results of this study. MQTLs names are shown on the right side of each chromosome, with black segments indicating their confidence interval. The genomic positions of the MQTL regions correspond to Table 3. The red bold markers in each chromosome represent location of significant SNP identified by GWAS. Ruler on the left side indicates the Mb distance.

Eleven of the 30 meta-QTLs (Table 3) were detected in two independent studies (MQTL2A-1, MQTL2A-2, MQTL2B-5, MQTL2D-2, MQTL2D-5, MQTL5D-1, MQTL6D-1, MQTL6D-2, MQTL6D-3, MQTL6D-4 and MQTL7D-1), seven meta-QTLs in three studies (MQTL2A-3, MQTL6A-3, MQTL6A-4, MQTL6B-1, MQTL7A-1, MQTL7A-2 and MQTL7A-3), six meta-QTL in 4 studies (MQTL2B-1, MQTL2B-3, MQTL2B-4, MQTL2D-4, MQTL6A-1 and MQTL6A-2), two meta-QTL in 5 studies (MQTL2D-3 and MQTL5D-2), two meta-QTL in 6 studies (MQTL1A-2 and MQTL2B-2), one meta-QTL in 7 studies (MQTL2D-1), and one meta-QTL in 8 studies (MQTL1A-1). Furthermore, genomic locations of detected MQTLs for tillering number that overlap with significant SNP markers from the GWAS results depicted in Fig. 7. five significant MTAs including IWA6592 (2A, 715,301,715 bp), IWB9024 (2A, 747,144,155 bp), IWB55568 (2D, 641,963,416 bp), IWB25250 (6A, 574,486,383 bp) and IWA1406 (6D, 463,447,036 bp) for the tiller number were located in the MQTL2A-3, MQTL2A-3, MQTL2D-4, MQTL6A-4 and MQTL6D-4 regions, respectively (Fig. 7).

## Discussion

In the present study, we evaluated the effects of two traits, total tiller number and fertile tiller number, in a collection of 92 landraces and cultivars of Iranian wheat. Significant variation in both traits appeared to be complementary between the genotypes and drought-stressed environments. In all tested environments, these traits decreased when drought stress occurred during the anthesis period. As Sareen et al.^26^ put it, the extent of the reduction in the tiller and fertile tiller numbers due to drought stress depends both on the magnitude of stress and the growth stage of the plant when stress occurs. However, the genotypes were insignificantly affected by stress and locations; the small magnitude of these effects were likely a result of low variations in genetic responses to the various stress conditions in the three tested environments (Shahed university research farm for two years and NIGEB field for one year), and due to stability of these genotypes in different environments.

A significantly positive correlations was observed between TTN and FTN in the normal and drought and all growing environments. Due to the importance of the relationship between tillering traits and other agronomic traits, such as grain yield and phenological characters, PCA biplots and linear correlation analysis also was performed (Supplementary Fig. S2). Between total tiller number and days to heading, days to flowering and days to maturity, there were significant positive correlations. Earlier reports from Mecha et al.^27^, Al Rabi^28^ depicted similar results. Begum^29^ and Qaseem et al.^30^ also reported that marker IWB44377 (which was associated with TTN and FTN in our study) had significant association with the days to heading and days to maturity traits, respectively. There was only a significant correlation between FTN and grain yield in normal conditions; this shows that increase in number of fertile tillers may result in proportionate enhancement in grain yield per plant. However, because of the complicated relationships between the majority of the traits (with each other and with yield), simple correlation coefficients may not provide a comprehensive information about the relationships between different traits, and it is critical to apply multivariate statistical methods, such as factor analysis, in order to better understand these relationships. Tillering capacity with moderate heritability is widely agreed to be the trait with the greatest effect on the yield potential of cereal^31^, even if there was no positive correlation between these in the initial analysis^32^. In the previous paper, we have reviewed this issue for some of these genotypes^33^ and found that, for instance, the first factor, which was named as yield components, was composed of some of the constituents of total tiller number, fertile tiller number, plant height, peduncle lenght, biological yeild and harvest index. Similar results were also observed in a study by Arminian et al.^32^.

GWAS is highly dependent on the presence of linkage disequilibrium in the population, although genetic drift, population structure, and natural selection have always been influential as well. Structure and PCoA analysis classified the 92 Iranian wheat genotypes into two groups with approximately 20 genotypes belonging to both groups. These intermediate genotypes were mostly the cultivars that could be placed in the landrace group since they had been selected from Iranian landraces. Based on recent investigations (Zarei Abbasabad et al.^34^, Alipour et al.^21^), high amount of genetic variability and diversity has been observed among Iranian landraces and cultivar wheat collected during different years, and in different geographical regions. These variations can be practical gene sources for use in breeding to deal with climate change challenges. The structure analysis in this study confirmed the clustering result of Alipour et al.^21^; our genotypes had similar stratification. They conducted a cluster analysis for 369 Iranian hexaploid wheat genotypes including 99 cultivars and 270 landraces using 16,506 GBS-SNP markers and reported that accession pedigree was the principal factor affecting the separation of Iranian cultivars. Most wheat cultivars originating from Iran or with one Iranian parent evidently vary from those originating from CIMMYT, resulting in a varied genetic makeup for Iranian wheat compared to CIMMYT wheat. This point could possibly justify the high-yielding cultivars derived from Iranian and CIMMYT wheat genotype crosses. For instance, the most widely planted cultivars in Iran, Parsi and Pishgam, have been obtained from a cross between CIMMYT and Iranian genotypes.

Similar to previous results reported by Berkman et al.^34^, Edae et al.^36^, and Al Rabbi^28^ maximum SNPs mapped to B-genome, followed by A genome and sequentially D-genome (as the youngest of these genomes in wheat formation history). It is presumed that the sequence polymorphism is a result of older genomes undergoing gene duplication and accumulating more mutations. While considerable early gene flow might have happened between *T. aestivum* and *T. turgidum* (AABB), it seems improbable between the *T. aestivum* and *Aegilops tauschii* (DD); otherwise, there could have been less sequence diversity in the D genome compared to B and A genomes^28,35^. As the results of present study showed, roughly twice the number of the SNPs mapped to D genome, mapped to the A or B genomes. While these results are in line with those of other studies^36,37^, they are contrary to some previous results^38,39^ that have shown that the number of SNP markers mapped to the B and A genome were five times greater than those mapped to the D genome. This difference suggests a comparatively high level of SNP variation in the D genome for Iranian wheat genomes when compared with other genotypes. As Jia et al.^40^ state higher variations in the D genome could prepare new useful and elite alleles that can control important agronomical traits to cope with global climate changes.

Varshney et al.^41^ and Wang et al.^42^ state that in QTL mapping approaches the two main factors involved in detecting significant MTAs are high values of heritability and abundance of polymorphic markers. However, Tavakol et al.^43^ indicated that a small population size is inevitably a limiting factor in detecting related loci with low effects. Despite the comparatively small size of our studied panel and the moderate value of heritability, the results of our survey seem reliable because the data were collected under six different conditions and also the BLUEs data based on these environments were more stable.

The outcome of GWAS was the identification of 24 significant MTAs (*P* < 0.05 after applying FDR-correction) for both traits in two or more environments; these results suggest pleiotropy, but may also suggest linkage. The SNPs significantly associated with TTN and FTN were determined on nearly all of the chromosomes tested, and were mainly distributed on chromosomes 1A, 2A, 2D, 6A, 6D, 7A and 7D (Supplementary Table S4). The genome-wide association study of Qaseem et al.^30^ showed that all the markers significant for tillers per plant, under a combined high temperature and drought regimes, were present on chromosomes 2A, 3B, 3D, 4B, 5A, 6A and 7D. Guo et al.^44^ identified different MTAs for tiller on chromosomes 1A, 2A, 3A, 4A, 5A, 6A, 7A, 1B, 2B, 3B, 4B, 5B, 6B and 6D and some of them were close to the associated SNPs in our study. The association study of Chen et al.^4^ employed a high-density 90 K SNP array to evaluate a panel of 205 elite winter wheat accessions. Their work demonstrated that 27 loci were associated with tiller number traits in different growth stages. Although no associations for the same SNPs were detected and the evaluated stages of the traits and the number of SNPs differ in this study, taken together, all of these loci harbor some tillering responsive genes that may play a key role in determining tiller-related traits.

In general, most MTAs are identified in a single environment and affect certain traits differently under different growing environments. As Chen et al.^4^ described, results in inconsistent association of markers or loci with specific characteristics if environmental conditions alter. This phenomenon has been witnessed on different markers that were putatively stress-specific, such as IWA6592 (Chromosome 2A), IWB53633 and IWB74344 (Chromosome 1A), which were associated only with TTN under stress condition. In contrast, the expression of a consistent MTA is less affected by environmental factors. An MTA stable across the different environments is of great value to marker-assisted selection (MAS) in breeding genotypes adapted to diverse ecological environments^4^. Overall, in this study, five MTAs (IWB44377, IWB39005, IWA5084, IWA4483 and IWB44155) were consistently associated with TTN or FTN in two water regimes environments (− log_10_ (*p*-value) ≥ 4.5), and the result considered relatively stable loci controlling tiller traits.

A pleiotropic locus influences the expression of more than one phenotypic trait and the loci controlling these traits can be in the same genomic position. Pleiotropic effects are especially beneficial in crop breeding, as they permit the breeder to choose multiple traits simultaneously^30^. In this study, pleiotropic MTAs (i.e., IWB44377, IWB39005) were identified which were associated with both TTN and FTN under all conditions. High phenotypic trait correlations (with *r*^*2*^ greater than 0.82 between TTN and FTN in all environments) might explain this pleiotropic effect. Mwadzingeni et al.^45^ said this is supported by the presence of different multiple MTAs in which one gene pleiotropically affects highly correlated characteristics and a common QTL often controls highly correlated traits. A review of other studies showed that Marker IWB39005 at 36.63 Mbp on Chromosome 2A was previously reported to be associated with tiller dry weight^44^. Marker IWB44155 at 323.74 Mbp on chromosome 7A (Table 1) which was associated with TTN under normal and drought stress treatment, associated with spike dry weight^46^, quality traits^47^ and resistance to *zymoseptoria tritici*^48^. Marker IWB44377 has significant association with days to maturity and grain yield traits in bread wheat cultivars reported by Qaseem et al.^30^. Genomic region (IWB11256) on chromosome 5D at 489.77 Mbp, associated with number of grains per ear (reported by Amer^49^) and anther extrusion (reported by Muqaddasi, et al.^50^). Furhrmore, Mohajeri Naraghi et al.^51^ reported one association i.e., IWA4483 for end-use quality. Zanke et al.^52^ also identified IWB28961 marker which was associated with thousend grain weight. A region (AX-95138710) near marker IWB55568, (on chromosome 2D) was reported in another study by Guo et al.^44^ and found to be significantly associated with spike length and total spikelet number. Ideally, the effects of such pleiotropic loci may not be affected by changes in the external environment. When breeding for broad adaptation, such loci or genomic regions could be valuable in gene introgression or breeding programs. Therefore, these MTAs could be employed to begin mining tillering genes via bioinformatics analysis and to develop cleaved amplified polymorphic sequence (CAPS) markers for MAS.

Meta-analysis method has been used to integrate the QTL data in order to clearly identify regions of the genome that are most frequently involved in trait variation and to narrow down the confidence interval of the QTLs. The results of the meta-analysis strongly depend on the quality of the studies have identified QTLs, quality of QTL projection and confidence intervals of QTLs^10^. In this study, based on the integration of a consensus map with 14,548 molecular markers and through meta-analysis, we combined 217 initial QTLs located on chromosomes 1A, 2A, 2B, 2D, 5D, 6A, 6B, 6D, 7A and 7D into 30 meta-QTLs. Intriguingly, we found that five significant MTAs i.e., IWA6592, IWB9024, IWB55568, IWB25250 and IWA1406 for the tiller number traits were located in the MQTL2A-3, MQTL2A-3, MQTL2D-4, MQTL6A-4 and MQTL6D-4 regions, respectively (Fig. 7). These MQTLs with relatively narrow CI had the highest number of QTLs for TTN and FTN traits and therefore considered as one of the most reliable chromosomal positions that can assist wheat molecular breeding. These results suggest that some MTAs identified in our study can serve as important MTAs for marker-assisted selection and gene cloning of important tiilering genes. QTL linkage mapping and association mapping are two effective strategies to identify the genes responsible for particular traits in crops. Both methods have particular advantages, such as higher statistical power and lower false positive rate for QTL mapping and relatively high mapping accuracy offered by association mapping^53^. Combining meta-analysis and association mapping can exactly identify suitable candidate genes involved in complex agronomic traits, such as grain yield, biomass, and phenology in wheat, barley and other close cereal species^12^.

## Conclusion

Overall, despite the moderate value of heritability and the fairly small size of the panel examined in this study, the results revealed 24 significant loci (FDR ≤ 0.05) associated with two traits: total tiller number and fertile tiller number under two irrigation regimes conditions at chromosomes 1A, 2A, 2B, 2D, 5D, 6A, 6B, 6D, 7A, and 7D. Among these loci, markers IWB44377 and IWB39005 were consistently associated with both TTN and FTN in all environments; thus, they could be used to develop CAPS markers. In addition, a meta-analysis of QTLs associated with TTN and FTN validated the GWAS results. The results of the current study, as well as the MTAs detected in the population, can add to the presently available genetic resources, gene pools, and candidate genes for wheat breeding. They can also provide evidence for further examinations and studies on the genetic bases of wheat adaptation under various climatic conditions both in Iran and other countries.

## Materials and methods

### Plant material and experimental design

The study employed a set of 92 Iranian hexaploid wheat genotypes (Supplementary Table S3) including 22 landraces and 70 cultivars, kindly provided by the Seed and Plant Improvement Institute (SPII) of Karaj, Iran. They were selected among 180 local bread wheat genotypes from diverse breeding programs and were assessed using a randomized complete block design with three replications from 2013 to 2015.

The study examined the results under two irrigation regimes, at two locations and in different years. The first irrigation regime was a hundred percent field capacity until harvest, and the second had no irrigation after anthesis. The two locations were the research farm of Shahed university, located at Shahr-e-Rey, 15 km southwest of Tehran (35°34′ N, 51°8′ E), 1,130 MASL (two years, 2013–2015), and a research field at National Institute of Genetic Engineering and Biotechnology (NIGEB) located at Vardavard, northwest of Tehran, 35°44′ N, 51°10′ E, 1,305 MASL (one year, 2014–2015). The temperature at the first research location varied from a minimum of -15° C to a maximum of 41 °C, and the climate was characterized by a mean annual precipitation of 224 mm (the highest of which is 49% in winter and 21% in spring). Whereas in the second research field, the minimum and maximum temperatures were − 20 °C and 42°C, respectively, with annual precipitation of 247.3 mm (the highest of which is 43% and 36% in winter and spring, respectively). In both research fields, sowing was done by hand in plots of four two-meter rows at 25 cm apart. All field plots were tilled before being sowed. Fertility was constrained by low organic matter and phosphorus contents, with application of 50 kg ha^−1^ of N, 50 kg ha^−1^ of P_2_O_5_ on the surface which was tilled into the soil before sowing.

### Trait phenotyping and data analysis

Five plants were randomly selected from each experimental unit, and the collected data were averaged and recorded for subsequent analysis. Later, the intended traits, total tiller number per plant (TTN) and fertile tiller number per plant (FTN) were evaluated. For all the environments (i.e., the two irrigation regimes at the Shahed university field for two years and at the NIGEB field for one year), the datasets were balanced; hence, the best linear unbiased estimates (BLUEs) equaled the arithmetic means across environments, places and irrigation regimes. Initially, separate analyses of data for each environment were conducted. A linear mixed model including the effects of places, genotypes, and replications was used to examine the data and BLUEs of the genotypes for each condition and across environments and the results served as input for the second step of analysis. The second step employed a linear mixed model that included the effects of G × E variance, calculated by the GenStat software package (14th edition):

$$y = \mu + {\text{ genotype }} + {\text{ environment }} + e$$

in which *μ* represents an overall mean, both environment and genotype are fixed effects, and *e* is a residual term.

To identify discriminating traits, principal component analysis (PCA) was carried out using the ‘factoextra’ package^54^ and the results displayed in a biplot. Pearson’s linear correlation was used to assess the strength and direction of association among the quantitative traits and the results were visualized using the ‘PerformanceAnalytics’ package implemented in the R enviroment.

### SNP genotyping

For this step, approximately 1.0 g of young wheat leaf tissue was collected from each of the genotypes before the elongation stage and the total genomic DNA was extracted, using the Cetyl Trimethyl Ammonium Bromide (CTAB) method. DNA dissolved in TE buffer was shipped to TraitGenetics company (GmbH, Gatersleben, Germany) for high-throughput genotyping using a set of 15 k Illumina Infinium iSelect SNP array (Illumina Inc). After filtering out SNPs with 10% missing data and 10% minimum allele frequency (MAF), a total of 6,349 SNP markers were determined based on the Chinese Spring reference assembly IWGSC RefSeqv1.0 (https://urgi.versailles.inra.fr/) and later included in analyses. Moreover, PowerMarker V3.25 software^55^ was employed to estimate the number of alleles for each locus, MAF and polymorphism information content (PIC) values.

### Population structure and genome-wide prediction

An analysis of the population structure for the association panel was estimated by a Bayesian model-based approach accomplished in STRUCTURE software V2.3.4 with 6,349 SNP markers located at least two cM apart in the genome. For each subpopulation value K, ranging from 1 to 8, STRUCTURE was run 10 times with a burn-in of 100,000 iterations and 50,000 iterations for the analysis. The inference of true K, using an ad-hoc statistic ΔK, was calculated based on the second-order rate of change in the log probability of data between successive values^56^. The results were processed using Structure Harvester web version v0.6.94 (https://taylor.biology.ucla.edu/StructureHarvester/). Principal coordinates analysis (PCoA) was used to examine the population structure of the panel for later use in GWAS, generated through the 6,349 SNP markers utilizing the Paleontological Statics (PAST).

Fixed and random model Circulating Probability Unification (FarmCPU) is a Genome Wide Association Study (GWAS) method, which utilizes both Fixed Effect Model (FEM) and a Random Effect Model (REM), iteratively, to control false positives. FarmCPU substitutes kinship with a set of markers associated with the causal genes to remove the confounding between kinship in a mixed linear model (MLM) and genes underlying interested trait^22^. in the present study, Genome-wide association scans for tiller number traits were performed by FarmCPU model on the BLUEs values calculated for each condition (normal and drought stress), as well as for the BLUEs values of all conditions (based on 6 environments) using R package ‘FarmCPUpp’^57^ to calculate P-values for Manhattan plots and Q-Q plots.

Because many MTAs were found, we selected an overall cutoff significance level of − log_10_ (*p-value*) ≥ 3.0, which means one false positive is expected in one-thousand events. A second, more stringent threshold was also included: false discovery rate (FDR) ≤ 0.05 threshold (− log_10_ (*p-value*) ≥ 4.5). Thus, SNPs with corrected *p-value* ≤ 0.05 were considered significantly associated with TTN and FTN traits. The GWAS results were illustrated in a circular Manhattan plot using the R package ‘SOFIA’^58^. All the mentioned packages were implemented in R environment version 3.5.3^59^.

### QTL projection and meta-analysis

A QTL dataset for the total tiller and fertile tiller (or spike) number on ten chromosomes (1A, 2A, 2B, 2D, 5D, 6A, 6B, 6D, 7A, and 7D) was compiled from a review of 30 independent studies published from 2002–2020. Those QTLs with available map positions, log of odds ratio (LOD) scores and *R*^*2*^ values were integrated for analysis. A new integrated consensus map (Supplementary Table S5) was created using data from two identified well-known genetic maps in wheat including Soriano and Alvaro^13^ and Maccaferr et al.^60^ with high-density markers using BioMercator V4.2. This map has 14,548 markers on 4,813.72 cM total length with the distance average of 0.33 cM. Projections of the QTLs’ positions were based on a simple scaling method between the interval of the QTL flanking markers on the original map and the interval between these markers on the consensus map. The confidence interval of 95% on the consensus map was estimated according to the empirical formula proposed by Guo et al.^61^. After projecting the QTLs, the Biomercator V4.2 software was used for QTL integration and to predict the location of meta-QTL(s). Using the approach of Veyrieras et al.^62^ BioMercator estimates the most likely assumption based on model choice criteria from AIC, AICc, AIC3, BIC and AWE. Based on the values for five models, the best meta-QTL model with the lowest value was considered the best fit. The probability of QTL for every segment of 0.5 cM on the consensus map was estimated following the approach described as ‘QTL-overview index’^25^.

## Supplementary information

Supplementary Tables.

Supplementary Figures.
