# Supplementary material for: Detection of genomic regions associated with tiller number in Iranian bread wheat under different water regimes using genome-wide association study
Source: Sci Rep. 2020 Aug 20;10:14034. doi: 10.1038/s41598-020-69442-9 (PMC7441066; doi:10.1038/s41598-020-69442-9)
Supplement: Supplementary file 2 — Supplementary Figures. [file 41598_2020_69442_MOESM2_ESM.docx]

|  |  |
| --- | --- |

**Figure S1.** Distributions of best linear unbiased estimator (BLUEs) for total tiller number and fertile tiller number traits in 92 genotypes grown under normal irrigation, drought stress and all environments. The boxes indicate mean (center), mean plus one standard deviation (top), and mean minus one standard deviation (bottom).

| 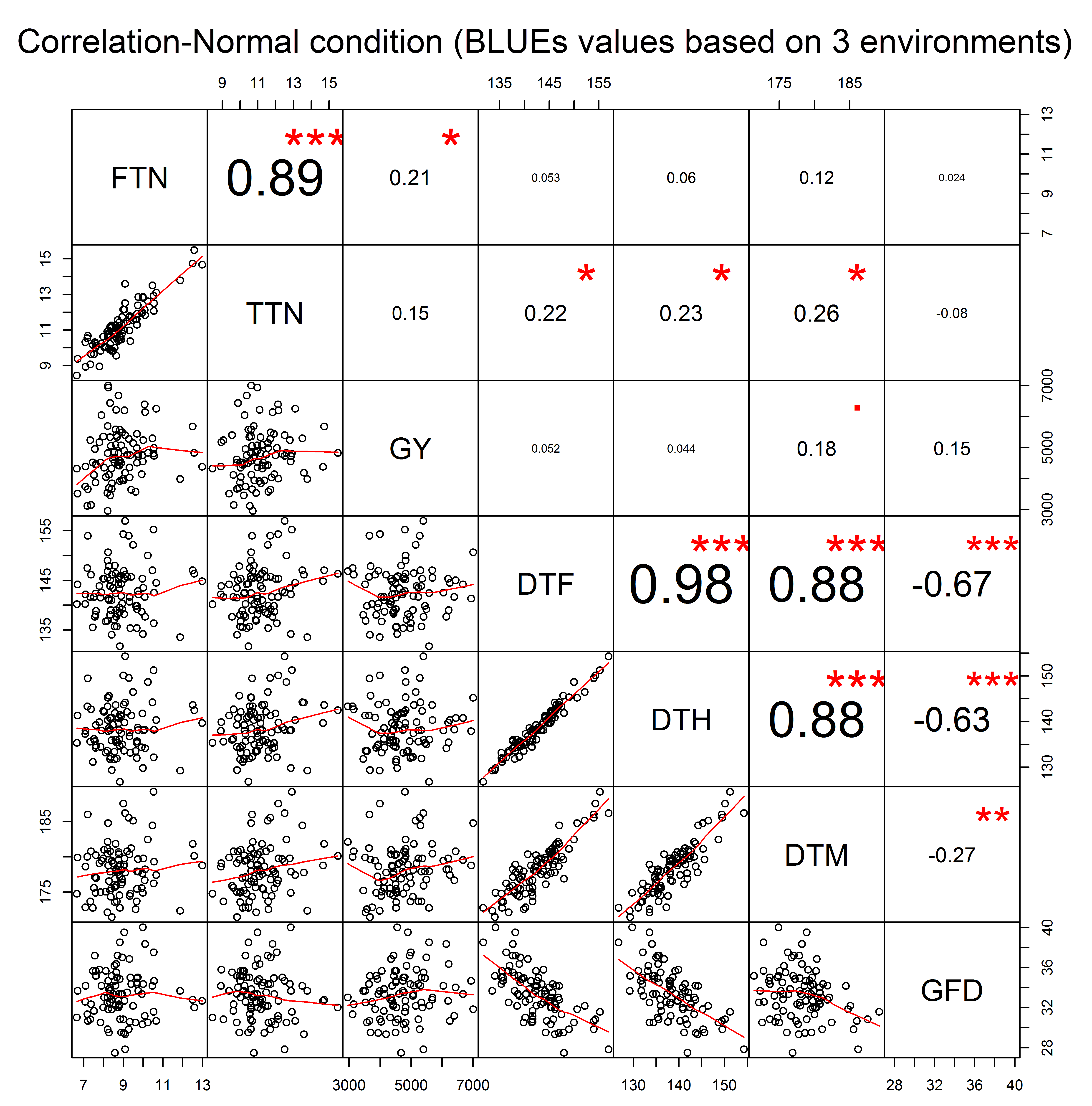 | 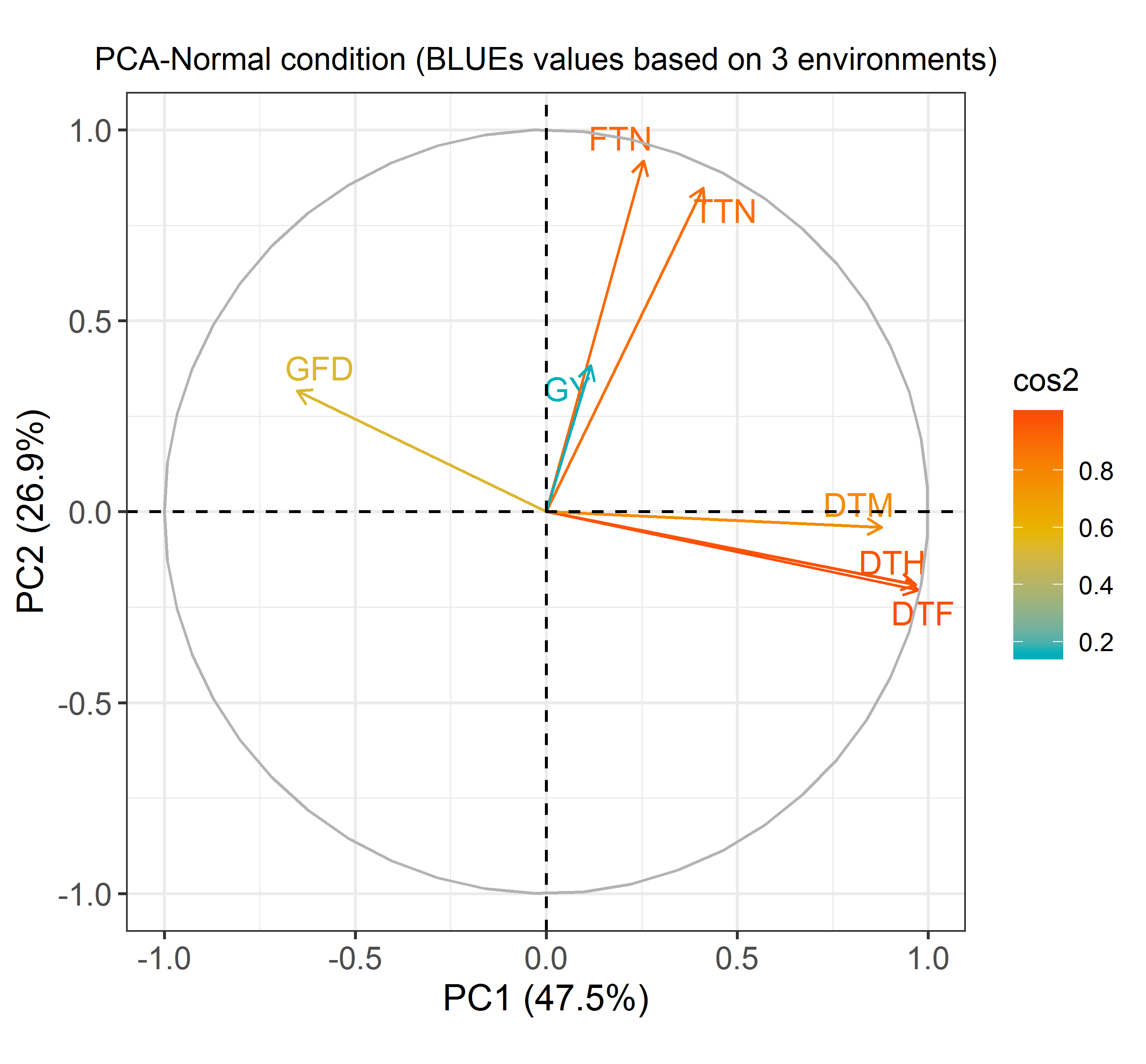 |
| --- | --- |
|  |  |
| 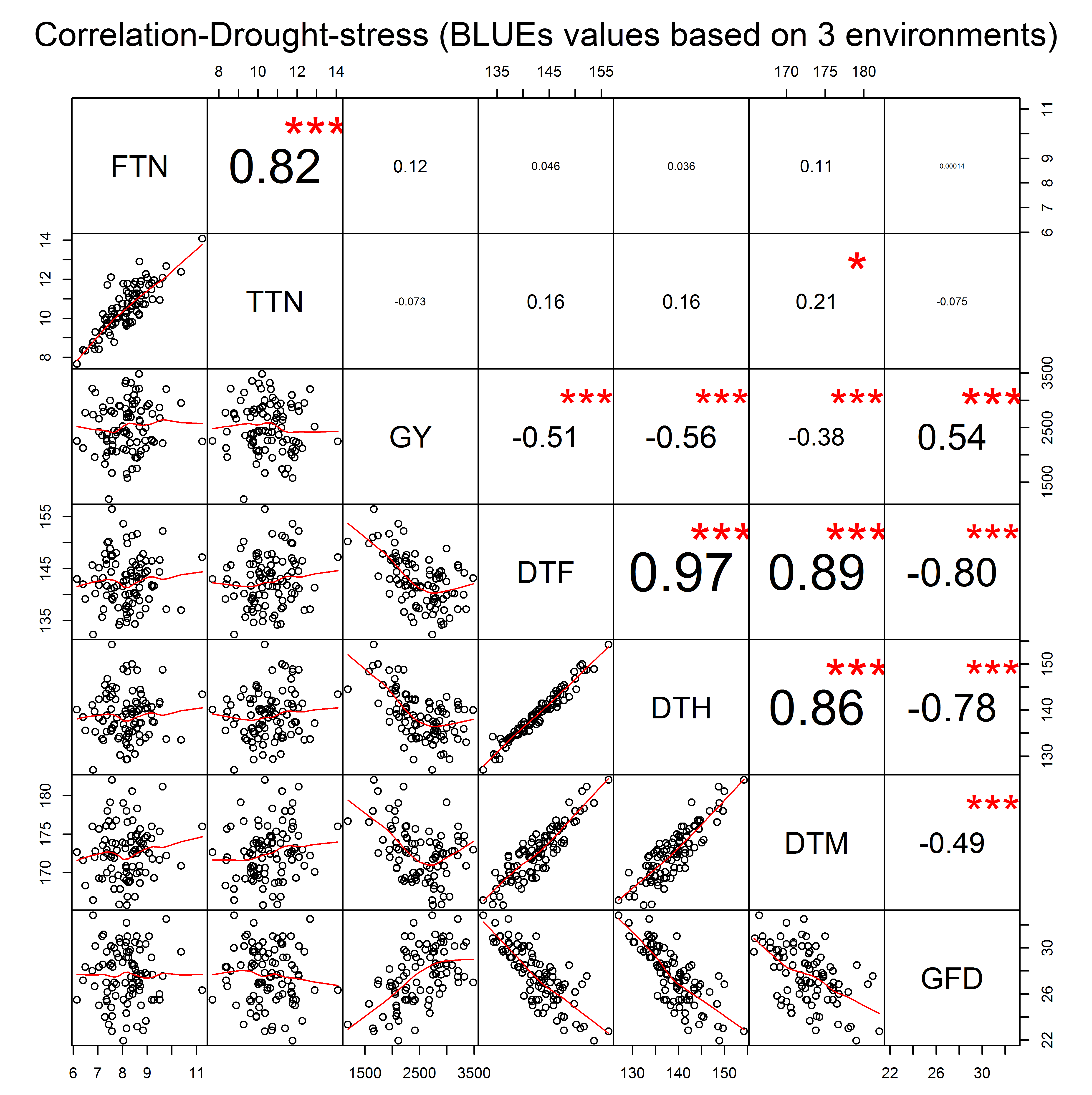 | 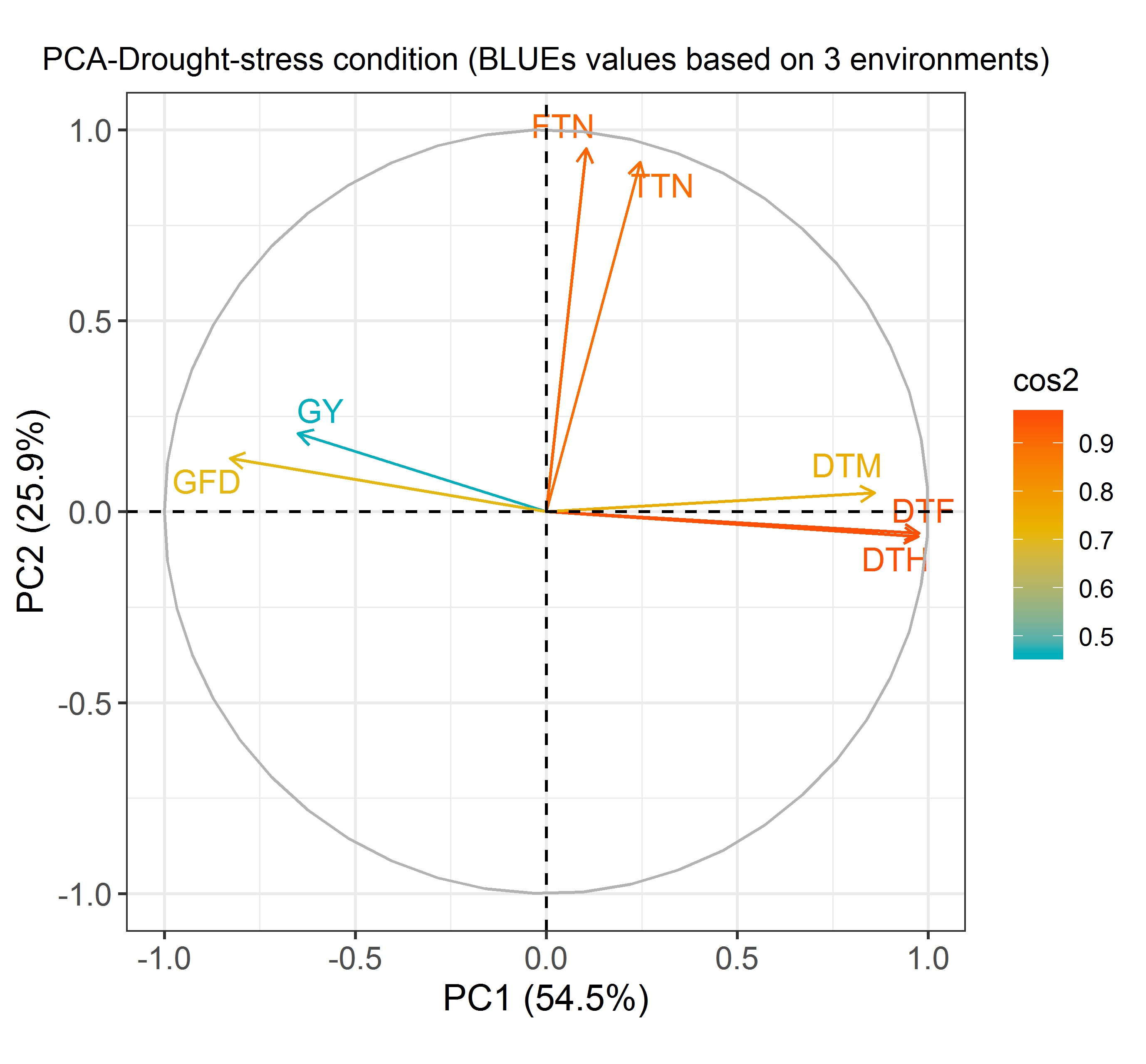 |
|  |  |
| 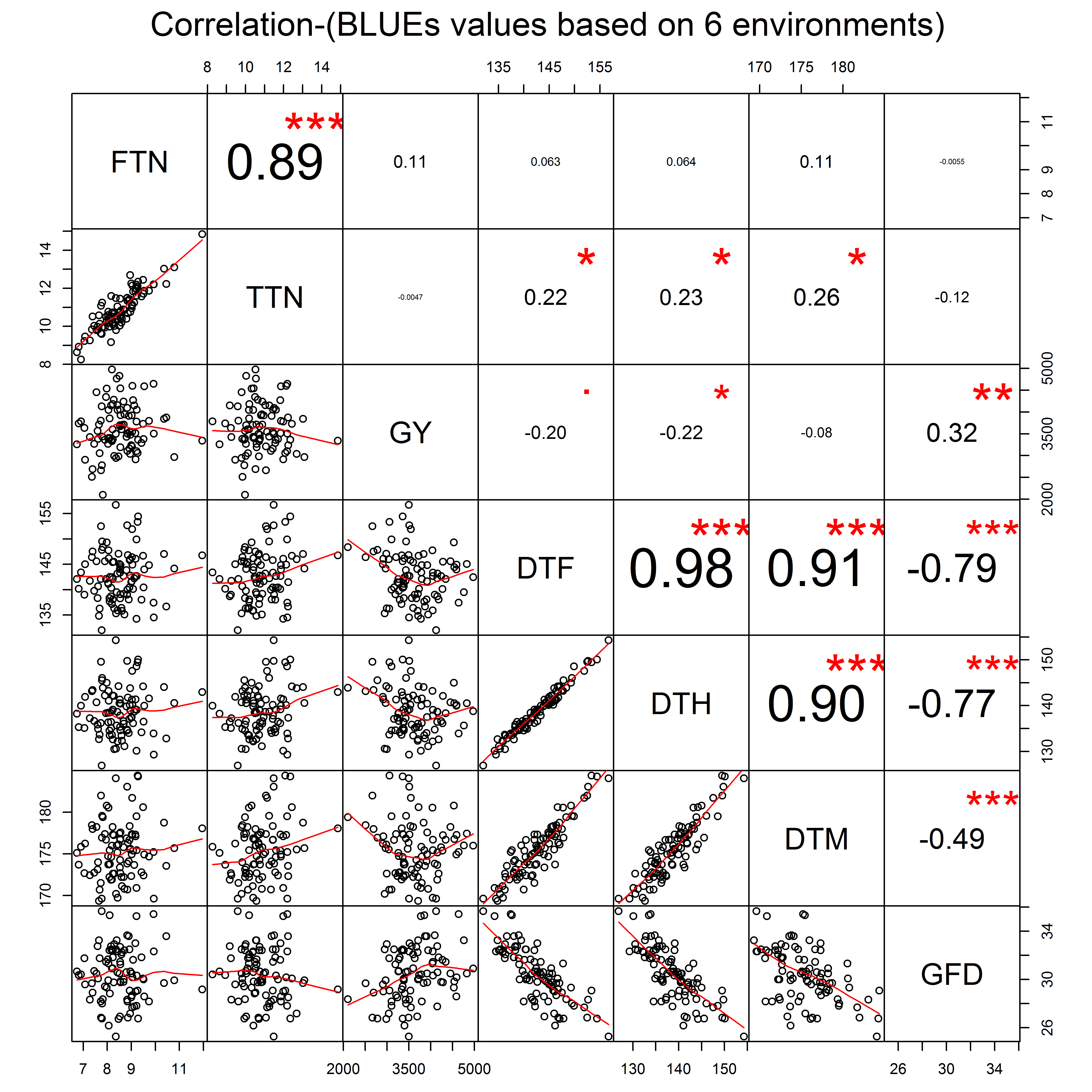 | 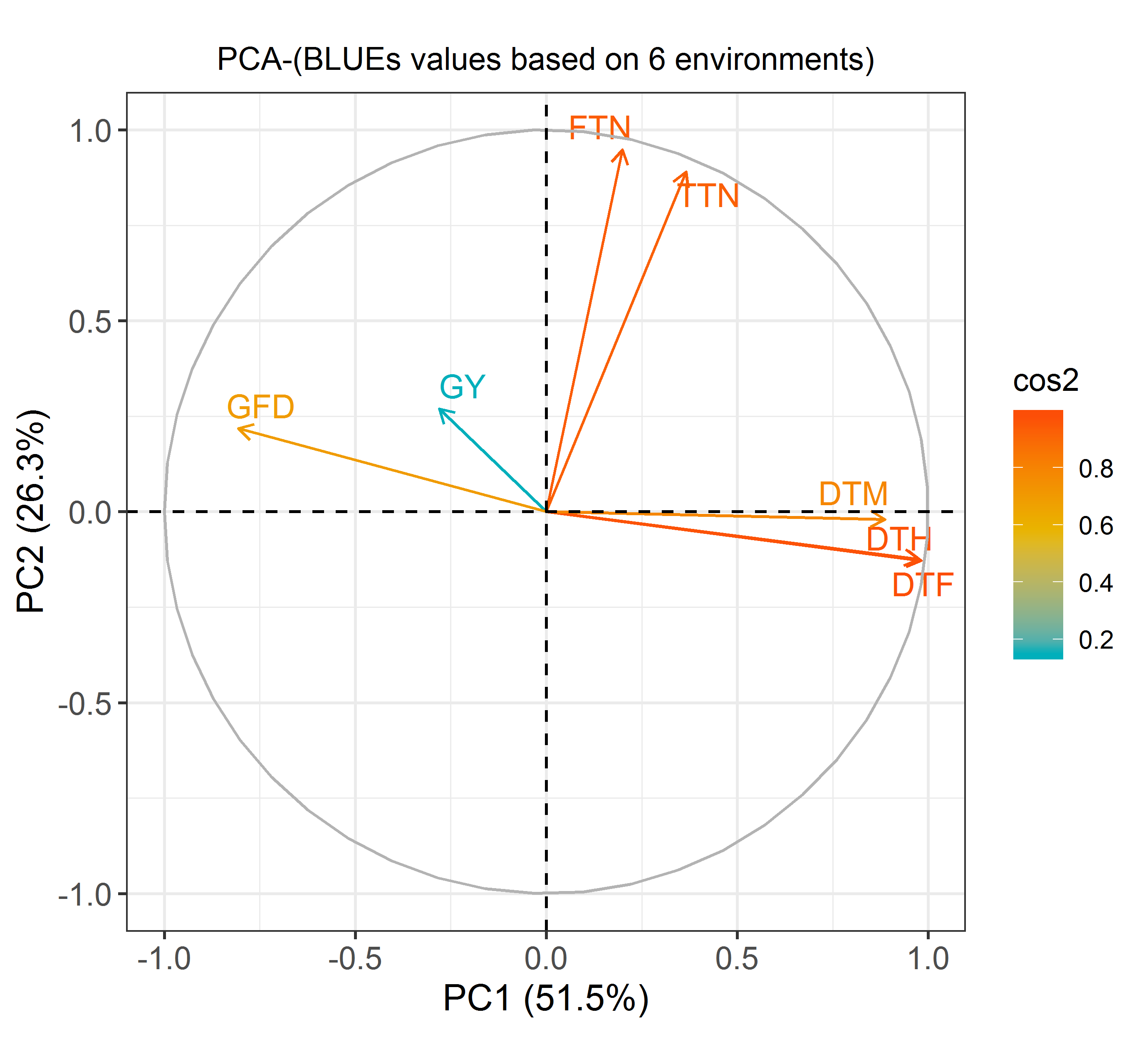 |

**Figure S2.** Principal component analysis and Pearson’s correlation coefficients between tiller traits, phonologic traits and grain yield based on BLUEs values in the normal, drought and all environments. FTN: Fertile Tiller Number, TTN: Total Tiller Number, GY: Grain Yield, DTH: Days to heading, DTF: Days to flowering, DTM: Days to maturity, GFD: Grain filling duration.
